# Lipid Metabolism and Cardiovascular Risk in HIV-1 Infection and HAART: Present and Future Problems

**DOI:** 10.1155/2010/271504

**Published:** 2010-10-31

**Authors:** Sara Melzi, Laura Carenzi, Maria Vittoria Cossu, Simone Passerini, Amedeo Capetti, Giuliano Rizzardini

**Affiliations:** ^1^1st Division of Infectious Diseases, “Luigi Sacco” Hospital, Via GB Grassi, 74, 20157 Milan, Italy; ^2^Clinical Pharmacology Unit, “Luigi Sacco” Hospital, 20157 Milan, Italy

## Abstract

Many infections favor or are directly implicated with lipid metabolism perturbations and/or increased risk of coronary heart disease (CHD). HIV itself has been shown to increase lipogenesis in the liver and to alter the lipid profile, while the presence of unsafe habits, addiction, comorbidities, and AIDS-related diseases increases substantially the risk of cardiovascular disease (CVD) in the HIV-infected population. Antiretroviral therapy reduces such stimuli but many drugs have intrinsic toxicity profiles impacting on metabolism or potential direct cardiotoxicity. In a moment when the main guidelines of HIV therapy are predating the point when to start treating, we mean to highlight the contribution of HIV-1 to lipid alteration and inflammation, the impact of antiretroviral therapy, the decisions on what drugs to use to reduce the probability of having a cardiovascular event, the increasing use of statins and fibrates in HIV-1 infected subjects, and finally the switch strategies, that balance effectiveness and toxicity to move the decision to change HIV drugs. Early treatment might reduce the negative effect of HIV on overall cardiovascular risk but may also evidence the impact of drugs, and the final balance (reduction or increase in CHD and lipid abnormalities) is not known up to date.

## 1. CHD and Infections

By the turn of the last century, medicine abandoned the link between infection, metabolic disorders, and atherogenesis because it did not fit in with the trends of the medical establishment convinced that chronic diseases such as heart disease must be multifactorial, degenerative, and noninfectious. Today, many patients with heart disease do not have the classic risk factors such as hypercholesterolemia, hypertension, smoking, or obesity; so it is not inconsistent that infection might underlie such damage. 

It is now generally recognised that cytokines, especially TNF, interleukin-1, and interleukin-6, which mediate the host acute-phase response to infection and inflammation, also mediate changes in lipid metabolism.

Several studies have addressed the possible role of infectious agents, both bacteria and viruses, in the pathogenesis of coronary heart disease and atherosclerosis [1, 2]. The concept of pathogen burden as a risk factor for CHD was first introduced by Epstein and his collaborators [3]. 

Both acute and chronic infections could play a role in the development of atherosclerosis and CHD. Acute infections (i.e., Coxsackie B viruse-induced myocarditis) could trigger acute cardiovascular events, and an acute respiratory infection during the 2 preceding weeks has been shown to be a risk factor for AMI in people with no history of classic risk factors [4].

Chronic infections may actively participate in the atherosclerotic process, leading to continuous low-level production of cytokines and thus to an atherogenic lipid profile, increasing triglyceride and total cholesterol, and lowering HDL concentrations [5]. Via their effect on lipid and glucose metabolism, chronic infections might also be related with obesity and the metabolic syndrome [6]. 

People infected with multiple pathogens, such as HSV-1, HSV-2, CMV, *H. pylori*, *C. pneumoniae,* and hepatitis A virus have high C-reactive protein rates as a marker of inflammation—a major relative risk factor for coronary artery disease [7]—and serological association between enteroviruses and human CHD has been suggested [8].

Finally, mycobacterial diseases share interesting connections to heart disease because the pathogenesis of tuberculosis depends on cholesterol and atypical tuberculosis caused elevation of C-reactive Protein, interleukin-6, and homocysteine 2 [9].

## 2. Dyslipidemia and HIV-1 Infection

### 2.1. In Vitro Studies

HIV-1 infection causes a specific pattern of dyslipidemia, resulting from a combination of increased production and decreased clearance of lipoproteins. Molecular mechanisms responsible for the numerous lipid-related disorders in HIV-infected individuals are not well understood. Adipose tissue hosts multiple cell types including monocytes, macrophages, endothelial and vascular smooth muscle cells. These immune cells are functionally active in the adipose tissue and produce numerous cytokines and other regulatory factors that influence lipid homeostasis, regulation of steroid hormones, prostaglandin, and fat-soluble vitamins. These factors also control storage of excess lipids and triglycerides (either normal and abnormal fatty acids) present in the circulation. Many infectious agents including HIV-1 have profound impact on adipocytes which become dysfunctional and cannot store most lipids—that is, triglycerides—properly.

Rasheed et al. [10] present the first direct evidence that HIV replication alone in human T-cells, without any influence of antiviral drugs or other factors, can stimulate the production of novel cellular enzymes and proteins that enhance fatty acid synthesis, increase the quantity of low density lipoproteins, secrete triglycerides, alter the lipid transport and metabolism, and oxidize lipids. This finding leads us to a new concept in HIV-1 pathogenesis. One of the most essential biological processes involved in dyslipidemia and lipodystrophy syndrome is the accumulation of lipids and disproportionate distribution of tissue-associated fats due to the enhanced fatty acid synthesis. Since kinases and enzymes activate most cellular functions including lipid synthesis, Rasheed first analyzed the functional significance of these proteins in HIV-infected cells in comparison with those expressed in the uninfected control cells.

They discovered that of the 18 differentially expressed proteins in HIV-infected cells, six enzymes/kinases were expressed exclusively in HIV-infected cells (CO3, P3C2B, KPCB, FAS, ACSL1, and GPX1) and one isomerase (PDIA3) was slightly downregulated after chronic HIV infection. They conclude that HIV-1 replication alone (i.e., without any influence of antiviral drugs or other human genetic factors) can induce novel cellular enzymes and proteins that are significantly associated with biologically relevant processes involved in lipid synthesis, transport, and metabolism (*P* = .0002–.01). This is the first direct evidence that HIV-1 modulates the production of proteins that are significantly involved in disrupting the normal metabolic pathways of lipids. However, the study presents several possible biases because it is not clear if the result would be the same with real cells; besides only X4 virus was studied which may not reflect the in vivo situation, mainly characterised by R5-tropic virus. Furthermore, polybrene was used to infect the cells, which artificially helps to bypass surface binding and entry by the virus.

Translational and clinical studies on the newly discovered proteins may now shed light on how some of these proteins may be useful for early diagnosis of individuals who might be at high risk for developing lipid-related disorders. The target proteins could then be used for future studies in the development of inhibitors for preventing lipid-metabolic anomalies. 

### 2.2. In Vivo Studies

Hellerstein et al. [11] measured de novo lipogenesis in three groups of patients (HIV-infected with history of weight loss, asymptomatic HIV-infected, and uninfected males) and found that hepatic lipogenesis was three to fourfold higher in the first group compared with seronegative controls (*P* < .05). The authors concluded that HIV infection was associated with abnormal fat anabolism.

Riddler et al. [12] evaluated changes in serum cholesterol associated with HIV infection and subsequent antiretroviral therapy, testing saved blood samples on 50 of 517 male seroconverters from the Multicenter AIDS Cohort Study. The outcome measures were changes in total cholesterol (TC), high-density lipoprotein cholesterol (HDL-c), and low-density lipoprotein cholesterol (LDL-c). These parameters were evaluated at six time points over a period of 12 years. After HIV seroconversion, they noted significant declines in TC, HDL-c, and LDL-c. Following the initiation of HAART, increases in TC and LDL-c to slightly above pre-seroconversion levels were seen. The authors concluded that HIV infection alone results in substantial decrease in TC, HDL-c, and LDL-c. They postulated that the posttreatment elevations in TC and LDL-c probably represent a return to preinfection lipid levels.

Moreover, many additional factors may influence atherogenesis and CVD, such as AIDS-related infections [13] or the fact that certain “classical” vascular risk factors are overrepresented in the HIV-infected population (i.e., cigarette smoking). On the other hand, recent studies have shown that, similarly to the observation made in the general population, HCV coinfection seems to be associated with lower blood levels of cholesterol in HIV patients receiving ART [14].

## 3. Cardiac Risk and HIV Infection 

Prior to the advent of HAART, infections and tumours were the prevalent causes of cardiovascular damage in HIV-1 infected subjects (pericarditis, miocarditis, endocarditis, and cardiac involvement in AIDS-related tumours). With the advent of HAART and the subsequent longer survival, the most common cardiovascular disease is ischemic disease [15].

However, it is not so clear to what extent antiretroviral therapy may increase or decrease the risk of cardiovascular disease (CVD). CVD, indeed, is more common in HIV-infected patients than HIV negative, mostly due to the higher prevalence of CV risk factors like metabolic abnormalities (dyslipidemia and insulin resistance) and drug consumption (tobacco, alcohol, cocaine) [16, 17]. Moreover, HIV infects smooth muscle cells *in vivo* promoting inflammation [18] and ACTG 5152s showed that untreated HIV infection is associated with endothelial dysfunction, and ART restores the endothelial damage [19, 20].

The difficulties in determining the cardiac risk among HIV-infected patients have largely been due to the lack of matched controls and to small sample sizes.

## 4. Cardiac Risk and Antiretroviral Therapy

Although some drugs may be more harmful than others in increasing CVD risk, it is now clear that any therapy is better than no therapy.

In the largest trial of intermittent treatment, the SMART study, patients assigned to the intermittent therapy arm had a higher incidence of cardiovascular disease [21]. In the STACCATO study, the interruption of therapy was associated with increases in markers of endothelial dysfunction and inflammation contributing to the development of cardiovascular disease [22].

When choosing the drugs to build an antiretroviral regimen, however, not every drug or class of drugs is equivalent in terms of impact on the CVD risk. 

The Data Collection on Adverse Events of Anti-HIV Drugs (D:A:D) investigators reported an increased relative rate (RR) of myocardial infarction (MI) with the cumulative use of protease inhibitors (RR per year of exposure, 1.16 [95% confidence interval {CI}, 1.10–1.23]) but not of nonnucleoside reverse transcriptase inhibitors (RR per year of exposure, 1.05 [95% CI, 0.98–1.13]) [23]. In a subsequent analysis focusing on nucleoside reverse transcriptase inhibitor use, an unexpected increased risk of MI was found with recent use of abacavir (RR, 1.90 [95% CI, 1.47–2.45]) and didanosine (RR, 1.49 [95% CI, 1.14–1.95]), but not with cumulative use [24].

Many trials and nonsponsored studies have assessed the efficacy and the lipid profile of all the approved antiretrovirals and nowadays the metabolic impact of a new drug is considered as important as its potency.

The STEAL study evaluated the incidence of CVD over 96 weeks in patients treated with abacavir plus lamivudine (ABC/3TC) versus tenofovir plus emtricitabine (TDF/FTC). Patients assigned to the ABC/3TC arm had significantly more cardiovascular events but unfortunately patients in ABC/3TC had a heavier history of risk factors than patients assigned to TDF/FTC [25].

The BICOMBO study, another randomized trial of switching to the thymidine analogue-sparing fixed dose combinations (FDCs), showed only one case of myocardial infarction in a younger population with a lower baseline Framingham score [26].

The ACTG 5202 study, a multicenter, randomized, blinded equivalence study compared antiviral activity, safety, and tolerability of ABC/3TC and TDF/FTC given with efavirenz or ritonavir-boosted atazanavir for the initial treatment of HIV-1 infection, followed for 96 weeks. A total of 1858 eligible patients were enrolled in the study from September 2005 to November 2007. This analysis includes data from 797 patients with a screening HIV-1 RNA level of 100,000 copies/mL or more. At week 48, fasting lipid levels had increased more in the patients who received ABC/3TC than in the patients who received TDF/FTC (median change in TC level: 34 versus 26 mg/dL, *P* < .001; HDL-c level: 9 versus 7 mg/dL, *P* = .05; and triglyceride (TG) level: 25 versus 3 mg/dL, *P* = .001). There was no significant difference between groups in the change in the ratio of TC: HDL-c (median, −0.2 for both groups; *P* = .50) [27].

The GEMINI study, a 48-week, multicenter, open-label study, confirmed the noninferiority of a TDF/FTC backbone plus saquinavir/ritonavir (SQV/r) 1000 mg/100 mg twice a day (*n* = 167) versus TDF/FTC plus lopinavir/ritonavir (LPV/r) 400 mg/100 mg twice a day (*n* = 170) in treatment-naive HIV-1-infected adults.

The rate and severity of adverse events were similar in both groups. There were no significant differences in the median change from baseline between arms in plasma lipids except for triglyceride levels, which were significantly higher in the LPV/r arm at week 48 [28].

KLEAN is an open-label, noninferiority study including 878 antiretroviral-naive, HIV-1-infected patients randomised to receive either fosamprenavir-ritonavir 700 mg/100 mg twice daily or lopinavir-ritonavir 400 mg/100 mg twice daily, each with the coformulation of ABC/3TC. At week 48, not only the noninferiority of fosamprenavir-ritonavir to lopinavir-ritonavir (95% CI around the treatment difference −4.84 to 7.05) was shown, but also the metabolic impact of the two regimens was comparable [29].

In the CASTLE study, once-daily atazanavir/ritonavir (*n* = 440) demonstrated similar antiviral efficacy as twice-daily lopinavir/ritonavir (*n* = 443), each combined with TDF/FTC. Treatment-related gastrointestinal adverse events were greater in patients taking lopinavir/ritonavir. Mean changes from baseline in fasting total cholesterol, non-high-density lipoprotein cholesterol, and triglycerides at week 96 were significantly higher with lopinavir/ritonavir (*P* < .0001) [30].

The ARTEMIS (AntiRetroviral Therapy with TMC114 ExaMined In Naive Subjects) trial, a randomized, open-label, phase III trial of 689 antiretroviral-naive patients with HIV-1 RNA at least 5000 copies/ml (stratified by HIV-1 RNA and CD4 cell count) receiving darunavir/ritonavir (DRV/r) 800/100 mg once daily or lopinavir/ritonavir (LPV/r) 800/200 mg total daily dose (twice daily or once daily) and TDF/FTC has disclosed 96-week data. DRV/r patients had smaller median increases in triglycerides (0.1 and 0.6 mmol/L, resp.; *P* < .0001) and total cholesterol (0.6 and 0.9 mmol/L, resp.; *P* < .0001) than LPV/r patients; levels remained below National Cholesterol Education Program cut-offs for DRV/r. At week 96, once-daily DRV/r was both statistically noninferior and superior in virologic response to LPV/r, with a more favorable gastrointestinal and lipid profile, confirming DRV/r as an effective, well tolerated, and durable option for antiretroviral-naive patients [31].

## 5. Pharmacologic Management of Dyslipidemia in HIV-Infected Subjects on HAART

The Infectious Diseases Society of America (IDSA) and the European AIDS Clinical Society (EACS) have provided guidance for the treatment of dyslipidemia and the reduction of cardiovascular risk in patients with HIV infection [32, 33]. 

In general, patients with dyslipidemia and HIV infection should be treated in a manner similar to the general population [47, 48]. Statins are recommended for patients with hypercholesterolemia, although in patients receiving boosted PIs, they must be used with caution due to the high risk of interactions. For example, pravastatin levels have been shown to increase by 81% when given with boosted darunavir [49], whereas all other PIs tend to lower its levels. Simvastatin and lovastatin should be avoided in patients on PI-based regimens given the large and unpredictable increases in area under the curve (AUC) [50, 51]. NNRTIs, namely, efavirenz, reduce the blood concentrations of simvastatin and atorvastatin [52]. Until now, pravastatin has been considered the “safest” to use in combination with boosted and unboosted PIs. Recently, also rosuvastatin levels have been shown to increase in HIV-seronegative subjects who are being treated with LPV/r [53]. In this study, the LDL-C responses to rosuvastatin during LPV/r coadministration were diminished despite the higher rosuvastatin levels observed, suggesting that the drug might be prevented from reaching the site of action (i.e., by efflux pumps). This observation has been confirmed in HIV-infected patients. The mean reductions in total cholesterol and LDL-C from baseline to week 4 on rosuvastatin 10 mg once a day were 27.6% and 31.8%, respectively [54]. A large randomized, double-blind, placebo-controlled, parallel group trial (*n* = 320) with clinical endpoints is currently ongoing in the UK, evaluating the efficacy of rosuvastatin in slowing the progression of the carotid intima-media thickness (C-IMT; as measured by the change in the mean IMT of the near and far walls of the distal common carotid arteries), as well as its impact on hs-CRP, total and fractionated cholesterol, tryglicerides, and apolipoproteins (APO A1, APO B and APO B/A1) over 2 years in HIV-infected patients stable on antiretroviral therapy for at least 12 months having a 10-year CVD risk lower than 20% (using the Framingham risk score) [55]. Ezetimibe, a new inhibitor of intestinal absorption of cholesterol, has been shown to be safe and effective in HIV-infected patients [56–58].

When hypertriglyceridemia alone is present, often as a quite common side-effect of boosted PIs, gemfibrozil or fenofibrate are indicated, with minor drug interaction risks [59, 60]. In some cases of mixed dyslipidemia, associating a statin and a fibrate may be the best approach for achieving NCEP ATP III lipid targets in patients with HIV infection, as shown in a recent study of pravastatin plus fenofibrate in HIV-infected subjects [61]. Study ACTG A5087 was a randomized trial of HIV-infected persons with combined hyperlipidemia who received fenofibrate or pravastatin monotherapy for 12 weeks followed by the association of both for up to 48 weeks in case of failure to meet NCEP goals for LDL-C, HDL-C, and triglyceride levels. Plasma levels of hs-CRP, lipoprotein particle and apolipoproteins A1/B, P-selectin (cell adhesion molecule), plasminogen activator inhibitor-1, and adiponectin were also monitored. The majority of subjects (60/74 chosen subjects) switched to dual therapy at week 12 (*n* = 32, pravastatin added to fenofibrate monotherapy and *n* = 28, fenofibrate added to pravastatin). Results showed that from baseline to week 12, adiponectin, apoB levels, and Apo B/A1 ratios all significantly decreased in the pravastatin and fenofibrate arms, whereas lipoprotein particle and Apo A1 increased significantly in the fenofibrate arm only (*P* = .01 for both measures). Combination therapy elicited improvements in lipid profiles without changes in the inflammatory and endothelial cell markers hs-CRP, plasminogen activator inhibitor-1, and *P*-selectin. From weeks 12–48, Apo B levels and Apo B/A1 ratios declined significantly in those subjects adding pravastatin to fenofibrate (*P* < .01 and *P* = .01, resp.), whereas adiponectin levels significantly decreased in both combination treatment groups. These investigators suggested that HIV infection or other comorbid infections may inhibit the anti-inflammatory effects associated with lipid-lowering agents in the general population.

A recent wide retrospective study on 829 patients with HIV infection and 6941 patients without HIV infection beginning lipid-lowering therapy for elevated low-density lipoprotein cholesterol or triglyceride levels from the Kaiser Permanente Cohort showed that overall the response to lipid-lowering agents in real life is worse in HIV-infected subjects. These had had smaller reductions in low-density lipoprotein cholesterol levels from statin therapy (25.6% versus 28.3%; *P* < .001), which did not vary by antiretroviral therapy class. They also showed smaller reductions in triglyceride levels on gemfibrozil compared to patients without HIV infection (44.2% versus 59.3%; *P*  _  .001), and reductions with gemfibrozil varied by antiretroviral therapy class (44.0% [*P*  _  .001] in patients receiving PIs only, 26.4% [*P*  _  .001] in patients receiving PIs and nonnucleoside reverse transcriptase inhibitors [NNRTIs], and 60.3% [*P*  _  .94] in patients receiving NNRTIs only). Rhabdomyolysis was diagnosed in 3 patients with HIV infection and 1 patient without HIV infection [62].

HIV-infected subjects often take fish oil supplements to control dyslipidemia, and some trials have confirmed their effect. In the A5186 prospective, phase 2 clinical trial study on 100 patients, twice-daily administration of fish oil supplement or once-daily fenofibrate reduced triglyceride levels by 283 mg/dL (46%) and 367 mg/dL (58%), respectively. Patients not responding to single medications were then treated subsequently with both agents showing a 65.5% reduction from baseline in triglyceride levels. With the combination therapy, 22.7% achieved triglyceride levels of ≤200 mg/dL, starting from a median level of 667 mg/dL [63]. Lower reduction in serum triglycerides emerged from another randomized study versus placebo, where fish oil supplements at week 8 reduced triglycerides by 25.5%  (*n* = 58) versus 1% in the paraffin oil control arm (*n* = 62). The subsequent open-label phase showed sustained efficacy through 16-week continued treatment with fish oil supplement, while those switched placebo to fish oil supplement had a 21.2% decrease in serum triglycerides [64]. 

## 6. Management of Dyslipidemia through Switch Strategies

Another widespread approach among physicians is the switch to antiretroviral regimens characterized by low metabolic impact. In recent years, among others, an Italian team of experts set out recommendations on the diagnosis, prevention, and treatment of cardiovascular complications in HIV-infected patients in the HAART era which included regimen switching rules to reduce the metabolic impact of drugs avoiding to expose the patients to virologic failure [65]. Indeed, particularly when substituting high-genetic barrier drugs such as boosted protease inhibitors, which can even work in cases of functional or true monotherapy, particular attention should be paid to the residual efficacy of the backbone (generally NRTIs), since the new drug would not have the same features, or, in the case of abacavir, might be directly affected by resistance to NRTIs. The most lipid-friendly drugs to switch to are abacavir, tenofovir, nevirapine, atazanavir, and most recently raltegravir and maraviroc. Unfortunately abacavir and maraviroc are currently charged with some suspicion of cardiac toxicity [66, 67]. Trials on the switch to abacavir have constantly showed significant reductions in total and LDL cholesterol as well as in tryglicerides [68–70], and whenever patients are accurately selected for the absence of NRTI resistance-associated mutations or of previous NRTI failure, this simplification does not imply a higher risk of viral rebound [71]. Unfortunately, some studies have included patients pretreated with dual NRTI regimens which, presumably due to baseline resistance, showed a higher rate of virologic failure (3 versus 0/52 [68] and 13% versus 10% [72]).

The simplification from an effective PI-based regimen to nevirapine generally proved to be safe and to improve the lipid profile, although sometimes some degree of hepatotoxicity was observed [73]. Lypodistrophy, however, is not reversed by the switch [74]. In a small 24-week insight study on 55 patients switched from a PI to nevirapine, the lipid profile improved with a significant reduction of apoB (from 0.98 to 0.92 g L(-1); *P* = .005) and triglycerides (from 2.02 to 1.66 mmol L(-1); *P* = .02). HDL cholesterol and apoA1 increased significantly (from 0.99 to 1.19 mmol L(-1); *P* = .001 and from 1.40 to 1.57 g L(-1); *P* < .001, resp.). The triglyceride enrichment of HDL significantly decreased after the replacement of PI by nevirapine (from 0.248 +/− 0.092 to 0.213 +/− 0.093; *P* = .003), leading to a longer HDL half life. All such changes were correlated with adiponectin levels [75]. This simplification approach has yielded satisfactory results on the lipid profile of children as well [76]. No excess failures were reported with nevirapine and in some cases this strategy maintained better virologic suppression at 12 months. [73] More recently, switch from efavirenz to nevirapine has also become a relevant hypothesis, since the former has always shown to be unable to revert PI-induced metabolic alterations [77] and has been associated with a certain degree of such side effects. Switching to nevirapine in such reports was associated with decreases in LDL-Cholesterol and increases in HDL, as well as with a reduction of neuropsychiatric effects [40, 78]. Viral suppression was maintained in all switchers and in one study also two initially viremic patients suppressed passing to nevirapine [78]. 

Switches to tenofovir from thymidine analogues [39], mainly stavudine [34, 79–81], have showed some good changes in the lipid profile. In particular, a study on 352 HIV-infected subjects followed for 48 weeks found a sustained reduction in median TC (−17.5 mg/dL; *P* < .001), LDL-C (−8.1 mg/dL; *P* < .001), and TG (−35 mg/dL; *P* < .001). HDL-C remained roughly unchanged (−0.8 mg/dL). Patients with baseline hyperlipidaemia showed greater reductions in LDL-C (−29 mg/dL; *P* < .001) and TG (−76 mg/dL; *P* < .001). The greatest TG reduction was observed in patients with severe hypertrygliceridaemia (−266 mg/dL; *P* < .001). The estimated 10-year cardiovascular risk decreased in all patients (*P* < .001) and to a higher extent in patients with baseline hyperlipidaemia. There was a trend towards reduction according to the use of lipid-lowering agents (11.6% to 9,9%; *P* = nonsignificant) [35]. However, the beneficial effect sought by many on mitochondrial damage and lipodystrophy is still controversial [38, 82], with the reduction of lactataemia in those patients who started from elevated serum lactates being the only constant finding. This switch provided good metabolic effect also in children although the data are biased by a contemporary substitution of efavirenz for PIs [36]. The switch from thymidine analogues to tenofovir is safe and no virological failures have been reported in the studies performed up to date. 

In recent years, the most studied simplification approach remains within the family of protease inhibitors and concerns atazanavir, a lipid-friendly compound. All the trials demonstrated a decline in total cholesterol and tryglicerides, in particular the large SWAN [37], SLOAT [46], and ATAZIP trials [44], but when a deeper look was brought, also HDL cholesterol tended to decrease [45] or remained stable [83], showing in this respect a difference from nevirapine simplification. Most recently it has been suggested that even the low dose ritonavir present in the boosted atazanavir-based regimens may do or maintain some metabolic harm and that the switch to unboosted atazanavir in virologically suppressed patients without resistance-associated mutations is associated with a more favourable lipid profile without risks of loosing the grip on HIV, and a large trial seems to confirm these data [84]. A small pilot study of 9 patients with dyslipidemia and insulin resistance, tested through hyperinsulinemic euglycemic clamp (insulin dose, 200 mU/m minute), showed in all a relevant increase in insulin sensitivity (+28%; *P* = .008) 12 months after the switch from a PI to boosted atazanavir [43]. However, two studies looked at endothelial function, through the measurement of the brachial artery flow-mediated dilation and at various inflammatory and oxidative stress markers in subjects switching to boosted atazanavir and unfortunately found no difference between baseline and week 12 and 24 values [85, 86]. Another study looking at age-related cardiovascular risk found a very limited impact by this approach, mainly due to the presence of other relevant and unchanged risk factors [87]. Overall, simplification with atazanavir does not alter viral response, some studies are showing slightly better control with the switch and others moderately favour the continuation arms.

An attempt to exclude nucleoside analogues by building a PI plus NNRTI regimen in the ACTG 5125 extension trial maintained viral suppression but worsened the lipid profile of the patients [42]. On the contrary, in the Switchmrk study, the randomized substitution of lopinavir/ritonavir with the integrase inhibitor raltegravir yielded an important drop in serum levels of total cholesterol (−12.6% versus 1.0% in those who did not switch), non-HDL cholesterol (−15.0% versus 2.6%), and triglycerides (−42.2% versus 6.2%) in 350 subjects in only 12 weeks [41]. Unfortunately, the inclusion of patients who did not have a fully effective NRTI backbone negatively impacted on the achievement of the noninferiority goal: at week 24, 84, 4% of the patients switched to raltegravir maintained a viral load <50 copies/mL versus 90,6% in the lopinavir continuation arm.

## 7. Conclusions

CVD risk in HIV infection results from the combination of the effects of host and viral factors and antiretroviral therapy. Antiretroviral therapy has been shown to reduce HIV-related inflammation, and in some studies also to reduce the impact of CVD. The most recent revisions of international guidelines for the use of antiretroviral agents have predated the decision to start therapy at earlier stages of the infection, to reduce non-HIV-related morbidity and mortality, mainly related to tumours and CVD. 

Understanding the differences between antiretroviral drugs with regard to lipid alterations, acceleration of atherosclerosis, and CVD risk is crucial to plan regimens that are to be maintained for decades.

The capacity to tailor antiretroviral therapy designed on individual characteristic and CVD risk profile awaits attention and ability to balance risk and benefit of antiretroviral therapy. The clinical impact of the QT prolongation by many antiretrovirals has not been fully elucidated up to date.

Finally, much work remains to be done in understanding the mechanisms of the risk posed by therapy and by HIV itself and the new antiretroviral drugs should not be considered safe *a priori*, as unexpected impact on CVD has emerged in some cases after many years. 

Recently, four new agents from four classes have been given approval in the US and Canada for use in HIV-infected persons. Two of these drugs (maraviroc and raltegravir) were the first of their respective classes (integrase inhibitors and CCR5 inhibitors) to be approved. In addition, another drug, rilpivirine, is in clinical trial Phase III.

Rilpivirine appears to be associated with lower rates of lipid elevations than efavirenz; although theoretically the inhibition of the CCR5 coreceptor seems protective of CVD, in the MOTIVATE study, the maraviroc arm was associated with more CVD events. 

Raltegravir has not shown up to date any particular impact on lipids or on CVD, while etravirine and darunavir also seem more lipid-friendly than many other agents. 

However, all these data are theoretical and do not fully answer to the fundamental question: in future years, starting therapy earlier, will the balance of CVD in HIV-infected patients be positive, due to the reduction of HIV-related damage, or negative, due to the cardiovascular impact of drugs that we may not fully appreciate today?

## Figures and Tables

**Figure 1:**
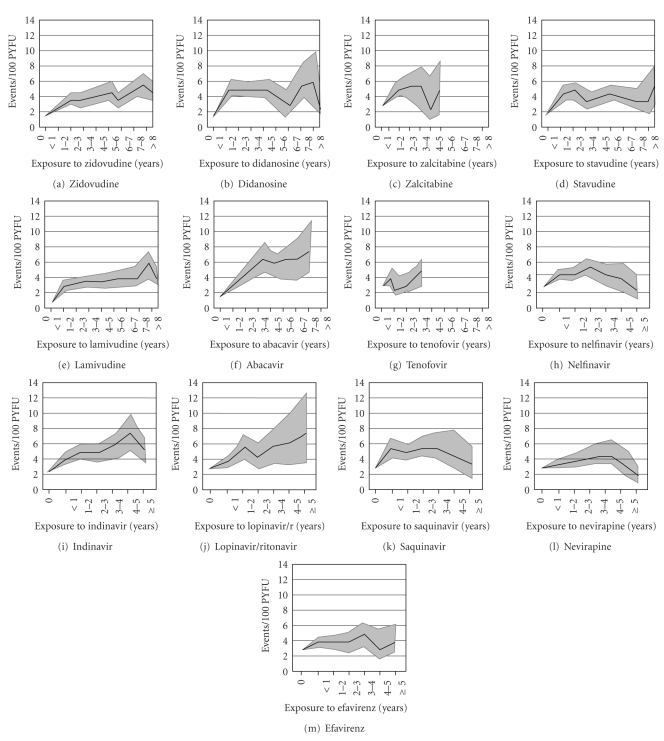
Incidence rates of myocardial infarction according to cumulative exposure to nucleoside/nucleotide analogues (*A–G*), protease inhibitors (*H–K*), and nonnucleoside reverse transcriptase inhibitors (*L, M*). The grey areas indicate the 95% confidence interval. PYFU, person-years of followup. Adapted from Worm et al. [24].

**Table 1:** Studies comparing the efficacy and the metabolic impact of different HAART regimens.

First author	Study Name	*N *	Study Design	F-up	Results
Cooper [25]	STEAL	350	Randomized to switch from current NRTI to TDF + FTC or ABC + 3TC FDC in HLA-B *5701^−^adults with plasma HIV viral load <50 copies/mL.	96 w	No significant difference for insulin sensitivity, total cholesterol (TC):HDL cholesterol (HDL-C) ratio, or lactate.
Martinez [26]	BICOMBO	335	Randomized to switch from 3TC-containing triple regimens to TDF + FTC (*n* = 166) or ABC + 3TC FDC (*n* = 167) in patients with <200 cp/mL for at least 6 months	48 w	One case of AMI Substudy 80 pts found no difference in IL-6, hsCRP, IL-10, MCP-1, TNFa, ICAM-1, VCAM-1, selectin, adiponectin, insulin and D-dimer
Sax [27]	ACTG 5202	1858	Naïve patients randomized to 4 once-daily antiretroviral regimens: ABC+3TC or TDF+FTC plus efavirenz or ritonavir-boosted atazanavir.	96 w	ABC+3TC versus TDF+FTC median change in TC: 34 versus 26 mg/dL, *P* < .001; HDL-C: 9 versus 7 mg/dL, *P* = .05; triglyceride (TG): 25 versus 3 mg/dL, *P* = .001.
Wamsley [28]	GEMINI	337	Naïve patients randomized to either SQV/r 1000 mg/100 mg twice a day (167) or LPV/r 400 mg/100 mg twice a day (170), plus TDF + FTC	48 w	No significant differences in the median change from baseline between arms in plasma lipids except for TG, significantly higher in the LPV/r arm
Eron [29]	KLEAN	878	Naïve patients randomized to either fosamprenavir-ritonavir 700 mg/100 mg twice daily or lopinavir-ritonavir 400 mg/100 mg twice daily, plus ABC/3TC	48 w	No significant differences between arms.
Molina [30]	CASTLE	883	Naïve patients randomized to atazanavir/ritonavir 300/100 mg once daily (440) opinavir/ritonavir 400/100 mg twice daily (443) plus TDF + FTC	24 m	Mean changes from baseline in TC, LDL-C, and TG were significantly higher with lopinavir/ritonavir (*P* < .0001).
Mills [31]	ARTEMIS	689	Naïve patients with HIV-1 RNA at least 5000 copies/ml (stratified by HIV-1 RNA and CD4 cell count) randomized to DRV/r 800/100 mg once daily or LPV/r 800/200 mg total daily dose (twice daily or once daily) plus TDF + FTC	96 w	DRV/r patients had smaller median increases in TG (0.1 and 0.6 mmol/L, resp., *P* < .0001) and TC (0.6 and 0.9 mmol/L, resp., *P* < .0001) than LPV/r patients

**Table 2:** Studies of switch towards more lipid-friendly antiretroviral regimens and variations in total and fractionated cholesterol and triglyceride blood levels.

Author	Year	Study drug	Switched drug	Design	Patients (*n*)	F-up (wks)	∆ TC (mmol/L)	∆HDL (mmol/L)	∆LDL (mmol/L)	∆ TG (mmol/L)
Negredo [34]	2002	Nevirapine Efavirenz	PIs	Randomized	77	48	*P* < .05 only Nev.	NA	NA	*P* < .05 only Nev.
Petit [35]	2004	Nevirapine	PIs	Observational	55	24	NA	+0,2 (*P* = .01)	NA	−0,36 (*P* = .02)
Ward [36]	2006	Nevirapine	Efavirenz	Retrospective	40	106	−0,18 (*P* < .05)	+0,05 (*P* < .05)	−0,25 (*P* < .05)	−0,70 (*P* < .05)
Parienti [37]	2007	Nevirapine	Efavirenz	Randomized	36	52	NA	*P* < .04	NA	NA
Gonzalez-Tome [38]	2008	Nevirapine	PIs	Pediatric, case-series	7	52	−0,7 (*P* = .09)	+0,5 (*P* = .03)	stable	NA
Katlama [39]	2003	Abacavir	PIs	Randomized	209	48	*P* < .001	NA	NA	*P* = .006
Keiser [40]	2005	Abacavir	PIs	Randomized	104	28	−0,82 (*P* < .001)	NA	−0,49 (*P* = .02)	−1,1 (*P* = .02)
Soriano [41]	2005	Atazanavir^a^	PIs	Randomized	189	48	−0,4 (*P* < .001)	NA	NA	−0,9 (*P* < .001)
Gatell [42]	2007	Atazanavir/r	PIs	Randomized	419	48	*P* < .001	*P* < .001	NA	*P* < .001
Mallolas [44]	2009	Atazanavir/r	PIs	Randomized	248	48	−0,4 (*P* < .001)	NA	NA	−0,5 (*P* < .001)
Llibre [43]	2006	TDF	Stavudine	Retrospective	352	48	−0,4 (*P* < .001)	NA	−0,2 (*P* < .001)	−0,4 (*P* < .001)
Schewe [44]	2006	TDF	Stavudine	Retrospective	66	130	−0,98 (*P* = .002)	NA	NA	NA
Madruga [45]	2007	TDF	Stavudine	Randomized	85	144	−0,6 (*P* < .0001)	NA	NA	−0,9 (*P* < .0001)
Valantin [46]	2010	TDF/FTC	NRTIs	Randomized	91	12	NA	NA	−0,4 (*P* = .03)	−0,4 (*P* = .03)
Eron [41]	2010	Raltegravir	Lopinavir/r	Randomized	707	12	−12,6% (*P* < .0001)	NA	−15% (*P* < .0001)	−42,2% (*P* < .0001)

^a^49 patients switched to unboosted atazanavir, 53 to atazanavir/r.

**Table 3:** Interactions between statins and protease inhibitors or nonnucleoside reverse transcripted inhibitors, from the DHHS Guidelines for the Use of Antiretroviral Agents in HIV-1-Infected Adults and Adolescents, revised Dec. 1, 2009. http://aidsinfo.nih.gov/contentfiles/AdultandAdolescentGL.pdf.

Interactions of statins with PIs			
Atorvastatin	All PIs	DRV/r ↑ 4 folds atorvastatin AUCFPV +/− r ↑ atorvastatin AUC by 130–153% LPV/r ↑ atorvastatin AUC by 488% NFV ↑ atorvastatin AUC by 74% SQV/r ↑ atorvastatin AUC by 79% TPV/r ↑ atorvastatin AUC by 836%	Use lowest possible starting dose with careful monitoring for toxicities or consider other HMG-CoA reductase inhibitors with less potential for interaction.
Lovastatin	All PIs	Significant ↑ lovastatin expected	*Contraindicated—do not coadminister.*
Pravastatin	DRV/r	pravastatin AUC ↑ 81%	Use lowest possible starting dose with careful monitoring.
	LPV/r	pravastatin AUC ↑ 33%	No dose adjustment necessary
		pravastatin AUC ↓ 47–50%	No dose adjustment necessary
Rosuvastatin	ATV/r	rosuvastatin AUC ↑ 213% and Cmax ↑ 600%	Use lowest possible starting dose with careful monitoring or consider other HMG-CoA reductase inhibitors with less potential for interaction.
	DRV/r, IDV +/− r, NFV, SQV/r	↑ rosuvastatin possible	
	FPV +/− r	No significant change	No dosage adjustment necessary
	LPV/r	rosuvastatin AUC ↑ 108% and Cmax ↑ 366%	Use lowest possible starting dose with careful monitoring or consider other HMG-CoA reductase inhibitors with less potential for interaction.
	TPV/r	rosuvastatin AUC ↑ 26% and Cmax ↑ 123%	
Simvastatin	All PIs	Significant ↑ simvastatin level NFV ↑ simvastatin AUC 505% SQV/r 400/400 mg BID ↑ simvastatin AUC 3,059%	*Contraindicated—do not coadminister.*

Interactions of statins with NNRTIs			

Atorvastatin	EFV, ETR, NVP	atorvastatin AUC ↓ 32%–43% with EFV, ETR	Adjust atorvastatin according to lipid responses, not to exceed the maximum recommended dose.
Fluvastatin	ETR	↑ fluvastatin possible	Dose adjustments for fluvastatin may be necessary.
Lovastatin Simvastatin	EFV	simvastatin AUC ↓ 68%	Adjust simvastatin dose according to lipid responses, not to exceed the maximum recommended dose. If used with RTV-boosted PI, simvastatin and lovastatin should be avoided.
	ETR	↓ lovastatin possible ↓ simvastatin possible	Adjust lovastatin or simvastatin dose according to lipid responses, not to exceed the maximum recommended dose. If used with RTV-boosted PI, simvastatin and lovastatin should be avoided.
	NVP	↓ lovastatin possible ↓ simvastatin possible	Adjust lovastatin or simvastatin dose according to lipid responses, not to exceed the maximum recommended dose. If used with RTV-boosted PI, simvastatin and lovastatin should be avoided.
Pravastatin Rosuvastatin	EFV	pravastatin AUC ↓ 44% rosuvastatin: no data	Adjust statin dose according to lipid responses, not to exceed the maximum recommended dose.
	ETR	No significant effect expected	No dosage adjustment necessary
